# Meta-analysis reveals conserved cell cycle transcriptional network across multiple human cell types

**DOI:** 10.1186/s12864-016-3435-2

**Published:** 2017-01-05

**Authors:** Bruno Giotti, Anagha Joshi, Tom C. Freeman

**Affiliations:** Systems Immunology Group and Developmental Biology Division, The Roslin Institute and Royal (Dick) School of Veterinary Studies, University of Edinburgh, Easter Bush, Edinburgh, Midlothian EH25 9RG UK

**Keywords:** Transcriptomics, Cell cycle, Systems biology, Network analysis, Meta-analysis

## Abstract

**Background:**

Cell division is central to the physiology and pathology of all eukaryotic organisms. The molecular machinery underpinning the cell cycle has been studied extensively in a number of species and core aspects of it have been found to be highly conserved. Similarly, the transcriptional changes associated with this pathway have been studied in different organisms and different cell types. In each case hundreds of genes have been reported to be regulated, however there seems to be little consensus in the genes identified across different studies. In a recent comparison of transcriptomic studies of the cell cycle in different human cell types, only 96 cell cycle genes were reported to be the same across all studies examined.

**Results:**

Here we perform a systematic re-examination of published human cell cycle expression data by using a network-based approach to identify groups of genes with a similar expression profile and therefore function. Two clusters in particular, containing 298 transcripts, showed patterns of expression consistent with cell cycle occurrence across the four human cell types assessed.

**Conclusions:**

Our analysis shows that there is a far greater conservation of cell cycle-associated gene expression across human cell types than reported previously, which can be separated into two distinct transcriptional networks associated with the G_1_/S-S and G_2_-M phases of the cell cycle. This work also highlights the benefits of performing a re-analysis on combined datasets.

**Electronic supplementary material:**

The online version of this article (doi:10.1186/s12864-016-3435-2) contains supplementary material, which is available to authorized users.

## Background

Cell division is a fundamental process common to all eukaryotic organisms and involves sequential duplication of the genome and daughter cell generation. These two events occur during S phase and M phase respectively, each preceded by a gap (G) phase, named G_1_ and G_2_, where cells grow in mass and prepare for the following phase. With the advent of genome-wide expression microarrays hundreds of cell cycle-regulated transcripts have been identified in yeast [1–5]. Likewise, in human cell lines several efforts have been put to define the cell cycle transcriptome. Among others, Whitfield and coworkers [6] synchronized HeLa cells both at G_1_/S transition, using a double-thymidine block, and at G_2_/M transition, using a thymidine-nocodazole block and identified 874 cell cycle-regulated genes. A later study on primary human foreskin fibroblasts identified 480 cell cycle-associated genes [7] after synchronization of fibroblasts both with a double-thymidine block and by serum deprivation. The latter synchronisation method forces cells to enter a quiescent state (G_0_), from which they can then re-enter proliferation as a cohort upon serum re-feeding [8]. More recently, the cell cycle transcriptome has been further characterized in two additional studies: one reported 1249 cell cycle-associated genes employing a human keratinocyte cell line (HaCat) which although immortalized is deemed to retain a normal cell biology [9], whereas the second study identified 1871 periodic genes in the osteosarcoma-derived cell line (U2OS) [10]. The studies mentioned above isolated periodic signals from the gene expression profiles, apparent when multiple cell cycle events are monitored, in order to identify cell cycle-associated genes. This is typically achieved by converting expression measurements for each gene into a wave function (Fourier transform), a method pioneered by Spellman and co-workers [1]. However, results from independent studies showed considerable discrepancies in the identity and size of the gene lists identified, with a large portion of genes being reported by only a single study [9, 11, 12].

Works on reconciling these diverse results have been carried out in budding and fission yeasts [11, 12]. These studies concluded that the primary cause of such discrepancies are not differences in experimental procedures nor in actual biological variation but rather in the analysis of the data. To date no similar studies have been performed on data derived from human cells. Cyclebase [13], a publicly available web resource provides a list of 378 human cell cycle-associated genes, but these have been derived from four experiments from only one study performed in human cells (HeLa cells) [6]. To address this gap we have re-analyzed publicly available expression data derived from four different human cell types using a correlation-based approach. This has enabled us to define conserved gene co-expression patterns associated with proliferation. Our analysis demonstrates that, as for yeast data, data interpretation is the primary reason for the discrepancies between previous results in defining a cell cycle gene set and that, contrary to what has been reported, the transcriptional network associated with the cell cycle is highly conserved across human cell types.

## Methods

### Description of cell cycle studies used for the meta-analysis

Gene Expression Omnibus (GEO) (http://www.ncbi.nlm.nih.gov/geo/) and ArrayExpress (https://www.ebi.ac.uk/arrayexpress/) data repositories were searched for microarray studies of the human cell cycle and filtered by array-based experiment. Four cell cycle studies were found, two on GEO (Acc. numbers: GSE52100, GSE26922) and two on ArrayExpress (Acc. numbers: E-MTAB-454, E-TABM-263). Raw data was available as cel files format with the exception of Grant et al. data, for which a preprocessed data matrix was instead available. Measurements in this dataset corresponded to the logged ratio of fluorescence intensities of the Cy3 (green) and Cy5 (red) fluorescent dyes. A brief description of the studies is summarized in Table 1.

**Table 1:** Description of cell cycle studies used for the meta-analysis

Cell line	Synchronisation method	Time points	Array platform	Study	Ac. Number	Raw data availability	Cell cycle genes (Entrez ID)
NHDF (primary fibroblasts)	Exp 1: Thy block Exp2: serum starvation	0 h–32 h, 2 h interval 0 h–32 h, 2 h interval	Affymetrix U133A 2.0	Bar-Joseph et al. 2008 [7]	E-TABM-263	yes	480
HeLa cells	Exp 1: Thy block Exp 2: Thy block Exp 3: Thy block	0 h–12 h, 2 h interval 0 h–12 h, 2 h interval 0 h–12 h, 2 h interval	Affymetrix HuGene 1.0st	Sadasivam et al. 2011	GSE26922	yes	_
HaCaT cells	Exp 1: Thy block Exp 2: Thy block Exp 3:Thy block	0 h–33 h, 3 h interval 0 h–33 h, 3 h interval 0 h–33 h, 3 h interval	Affymetrix HG-U133	Diaz et al. 2013	E-MTAB-454	yes	1249
U2OS cells	Exp 1:Thy block Exp 2:Thy block Exp 3:Thy block Exp 4:Noc block	0 h–46 h, 2 h interval 0 h–38 h, 2 h interval 0 h–46 h, 2 h interval 0 h–44 h, 2 h interval	Agilent Oligonucleotide arrays	Grant et al. 2013 [10]	GSE521000	no	1871

### Data processing

Three tests were performed to assess the array data quality from each study: 1) boxplots and histograms, to spot anomalous signal distribution and/or intensity, 2) pseudo-images of the arrays to identify spatial artifacts and 3) sample correlation matrix to identify low-correlated samples not associated with a different biology. After poor quality array removal, each sample set was normalized separately using robust multiarray averaging (RMA) normalization, a standard method for normalizing microarray data which implements background noise adjustment, quantile normalization and probe intensity summarization [14]. Next, probe sets were annotated with Entrez gene identifiers (Entrez IDs). Ambiguous probe sets mapping to multiple gene identifiers were removed. Quality control (QC), normalization and probe sets annotation was performed in R environment using a range of Bioconductor packages. Samples for each study were further examined after normalization by principal component analysis (PCA).

### Batch correction

Datasets were bound together using Entrez IDs as reference. The unified dataset contained 11,693 Entrez IDs and 159 samples. To adjust for different average intensities across datasets we applied ComBat [15], a widely used batch effect correction algorithm. Batches were manually numbered according to the study and then the algorithm was run in R environment.

### Cluster analysis

Cluster analysis was performed with BioLayout *Express* ^3D^ [16]. This tool allows the conversion of a data matrix into a correlation matrix by calculating Pearson correlations between every transcript to every other transcript measurement. Following the selection of a correlation threshold value, the correlation matrix is then rendered as a weighted undirected network, where nodes represent transcripts and the edges between them the correlation coefficients. A network clustering algorithm (MCL) is implemented within the tool to identify highly connected cliques of nodes within the network that represent genes with a similar expression profile [20]. Once the algorithm is run, clusters are color-coded and numbered according to their size in a descending order. Data was imported into BioLayout *Express* ^3D^ after converting the text file into an ‘.expression’ file. Measurements were anti-logged before the calculation of correlation matrix. The correlation cutoff threshold was set to *r* ≥ 0.60 and signal with a coefficient of variance lower than 0.18 was removed. The MCL inflation value (MCLi) of the cluster algorithm, controlling the granularity of the clusters, was set to 1.4 and the pre-inflation value was set to 2.0. Further sub-clustering of cell cycle-related clusters was obtained with MCLi at 2.3 for cluster 4 and 4.2 for cluster 6. Minimum cluster size was set to 5. Clusters of gene expression were then visually inspected. Specifically, we searched for clusters of genes whose average expression increased with a particular phase of the cell cycle across all datasets.

The clusters profiles are calculated as the average of the z-score of all the genes within the cluster. The z-score is defined as:

$$ z=\frac{x-\overset{\_}{x}}{sd} $$

where *z* is the z-score, *x *is the value of the given gene and *x* is the me﻿an of the ﻿values for the given gene.﻿

### ﻿Gene ontology enrichment analysis

Enrichment analysis was performed with Database for Annotation, Visualization and Integrated Discovery (DAVID) (Version 6.8 Beta), a web-based tool for Gene Ontology enrichment analysis (http://david.abcc.ncifcrf.gov/) [18]. Gene symbol lists were uploaded and analysed using Functional Annotation Clustering only for GO Biological Process annotation (GO_BP). Representative GO Biological Process terms selected from the top significantly enriched clusters were reported in figures. The Benjamini corrected *P*-values were used.

### WGCNA analysis

The R package “WGCNA” [17] was used to perform weighted gene co-expression analysis (WGCNA). Before construction of the adjacency matrix a soft threshold (β) was set by inspection of plots generated after calling the function *pickSoftThreshold*. The soft threshold was set to 6 as this value represented the point at which the Scale-Free Topology (SFT) Index as a function of the Soft Threshold reached saturation. Modules were generated after calling the function *blockwiseModules*. Arguments of this functions were kept as default. To run GO enrichments analysis the function *GOenrichmentAnalysis* was called returning the 10 most significant GO terms for each module. Benjamini-corrected *P*-*values* were used.

## Results

### Incongruences in previous cell cycle lists

Four previous cell cycle studies [6, 7, 9, 10] identified gene sets with periodic expression ranging from 480 cell cycle genes in fibroblasts to 1871 in U2OS cells. Grant et al. noted large differences between the gene lists (Additional file 1: Figure S1A) and phase assignation of the cell cycle genes based on their peaking times exhibited further incongruences. Three studies identified five different phases, namely: G_1_/S, S, G_2_, G_2_/M and M/G_1_ while in Bar-Joseph data S phase was not assigned. Percentage and number of genes assigned to the phases varied greatly across studies. Bar-Joseph et al. assigned 43% of the genes (221 genes) to G_2_/M phase while Grant et al. 21% (598 genes) to the same phase (Additional file 1: Figure S1B). Similarly, G_2_ phase accounted for 6% (29 genes) of the Bar-Joseph gene list and it comprised 21% (203 genes) of Peña-Diaz list. Only 18 genes were annotated as G_2_/M and 16 genes as G_1_/S consistently across all four studies while for the other phases (S, G_2_, M/G_1_), not even a single gene was identified by all studies (Additional file 1: Figure S1C). We therefore set out to perform a systematic re-analysis of the human core cell cycle transcriptome.

### Data processing and generation of a clustered network graph

**Fig. 1:**
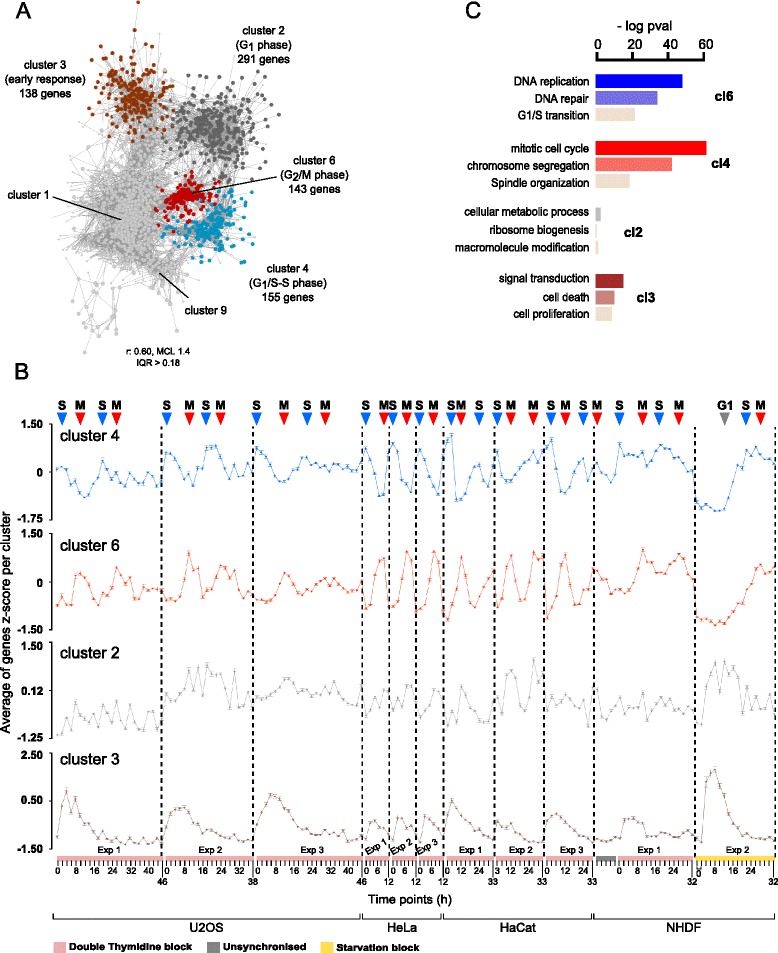
Cluster and GO enrichments analysis (**a**) Graph based on correlation of gene expression highlighting 4 of the most relevant clusters generated by the MCL. Clusters 4 and 6 represented the conserved core cell cycle signature from G_1_/S transition to mitosis. (**b**) Average expression profiles of the z-score for each gene within the clusters. *Error bars* represent standard errors. *Dashed lines* separate each experiment. Time points, number of experiments and cell types are specified on the x axis. Method of synchronisation is specified by color-coded bars above time points (see legend at the bottom). S, M and G1 phase events are highlighted on the *top* of the figure. (**c**) *Barplots* of three significant GO Biological Process terms after enrichment analysis using DAVID Functional Annotation Chart on the genes found in the respective clusters. Values are negative logarithms of the corrected *p*-values (Benjamini)

Data was collected from four microarray gene expression studies [7, 9, 10, 19] generated from four human different cell types: NHDF (primary fibroblasts), HeLa (cervical cancer cell line), HaCat (immortal keratinocytes), and U2OS (osteosarcoma cells), respectively. Low quality arrays were discarded by performing a number of QC metrics (see Methods) (Additional file 1: Figure S2–S5). Each sample set was then normalized separately using RMA normalization and data was further investigated using PCA plots to assess the presence of subtler batch effects and further samples were discarded (Additional file 1: Figure S2–S5). We next mapped probe sets from each dataset to Entrez IDs which were then used as reference to generate a collated dataset of 11,693 unique Entrez IDs and 159 samples. As the average intensity of each sample set was variable (Additional file 1: Figure S6A) we used *ComBat*, a batch correction algorithm which uses Empirical Bayes models to adjust for batch effects in the data (Additional file 1: Figure S6B). A correlation network from this data was reconstructed using BioLayout *Express* ^3D^_._ [16] After selecting for genes with Pearson’s correlation coefficient (r) ≥ 0.60, which was high enough for correlation not to occur by chance (Additional file 1: Figure S7A), the resulting network contained 3157 nodes (genes) connected by 21,858 edges (correlations). The network was then clustered using the MCL cluster algorithm [20] generating 68 different clusters of which six are here reported as showing reproducible pattern of expression across all the cell types and/or including relevant biology (Fig. 1a). Other clusters included noisy expression patterns which did not reproduce across samples or reflected artifact expression (Additional file 1: Figure S7B). Cluster 11 for example showed a sharp peak in a single sample not seen in the replicate samples and was therefore not considered for further analysis (Additional file 1: Figure S7C).

### Clusters with G_1_/S-S and G_2_-M phase specific gene expression

Genes in clusters 4 and 6 were maximally expressed during S and M phases consistent across all cell line assessed (Fig. 1b, blue and red profiles). The expression of genes in cluster 4 was up-regulated upon release from the double-thymidine block (which synchronises cells at the G_1_/S transition) and raised approximately 16 h after serum-refeeding in the serum starvation experiment in fibroblasts (Fig. 1b, blue profile). This is in agreement with an increased enrichment of S phase-related genes in human fibroblasts reported previously [21–23]. Conversely, cluster 6 genes exhibited low expression upon release from the thymidine-block followed by up-regulation at around 8–12 h and after around 24 h in the starvation experiment (Fig. 1b, red profile). Gene Ontology enrichment analysis of the 155 genes in cluster 4 demonstrated a highly significant enrichment for biological processes linked with S phase including *DNA replication* (*P* = 1.58 10^−55^), *DNA repair* (*P* = 1.31 10^−39^) and *G1/S transition* (*P* = 4.3 10^−26^) (Fig. 1c, blue barplots). The 143 genes found in cluster 6 were instead highly enriched for mitosis-related biological processes such as *mitotic cell cycle* (*P* = 3.46 10^−72^), *chromosome segregation* (*P* = 1.29 10^−48^) and *spindle organization* (*P* = 4.47 10^−22^) (Fig. 1c, red barplots).

Genes in cluster 4 included several factors involved in DNA replication such as various polymerases (*POLA1*, *POLA2*, *POLD1*, *POLD3*, and *POLE2*), proliferating cell nuclear antigen (*PCNA*), cell division control protein (*CDC6*) and other protein complexes necessary to initiate DNA synthesis e.g. members of the DNA replication complex (*GINS2*-*4*), members of the minichromosome maintenance complex (*MCM2-7* and *10*), and the replication factor complex (*RFC*s). DNA repair and DNA damage factors known to cooperate in DNA replication were also identified including Fanconi anemia complex components (*FANCE*, *FANCG*, *FANCI*, and *FANCL*), RAD complex components (*RAD51*, *RAD51AP1* and *RAD54L*) and Breast cancer type 1 susceptibility protein (*BRCA1*). Importantly, genes known to regulate G_1_/S transition including cyclins E (*CCNE1* and *CCNE2*), M phase inducer phosphatase 1 (*CDC25A*) and Cell division control protein 6 homolog (*CDC6*) belonged to cluster 4. Genes in cluster 6 included several G_2_ and mitotic regulators such as mitotic checkpoint serine/threonine-protein kinase (*BUB1*), cyclin-dependent kinase 1 (*CDK1*), a master cell cycle regulator, cyclins A and the two isoforms of cyclin B (*CCNA2*, *CCNB1*, *CCNB2*) and M phase inducer phosphatase 2/3 (*CDC25B* and *CDC25C*). Various genes involved in kinetochore formation (*CENPA*, *CENPE*, *CENPF*, *CENPI*) and several motor proteins members of the kinesin-like proteins (KIFs) known to participate in chromosomal and spindle movements during mitosis [24] also belonged to this cluster. Clusters 4 and 6 together accounted for 298 genes which exhibited up-regulation associated with S phase and mitosis across all the four cell lines examined. This number is three fold higher than that previously found to be representing the core cell cycle signature across the human cell lines investigated [10].

### Sub-clustering of clusters 4 and 6 allows more specific cell cycle phase association

**Fig. 2:**
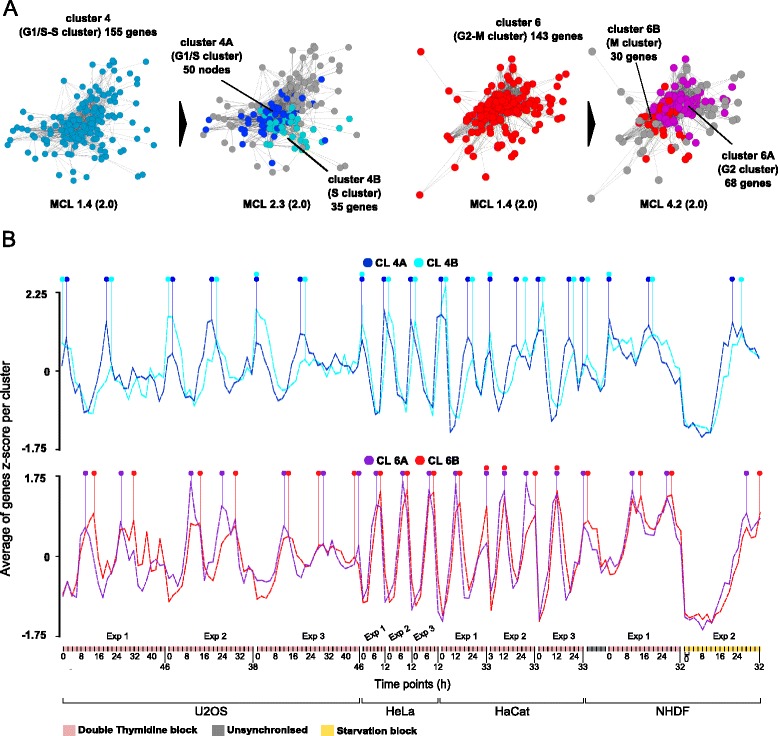
Separation of cluster 4 and 6 in multiple sub-clusters. (**a**) When the clustering algorithm inflation value was increased to 2.3, cluster 4 separated in clusters 4A and 4B, indicative of G_1_/S and S phase (*left*). The algorithm generated other 3 clusters which were omitted for clarity. Similarly, cluster 6 split in clusters 6A and 6B when inflation value was set to 4.2 representing G_2_ and M phase respectively (*right*). (**b**). Comparison of expression profiles of clusters 4A and 4B (*top*) and clusters 6A and 6B (*bottom*). A slight shift in the peaks of expression can be observed for both pair of clusters which is almost entirely consistent across data (see *dots* on top of expression profiles)

As in the previous cell cycle studies genes were assigned to at least four different cell cycle phases, we investigated if more detailed phase-specific gene networks could be identified from cluster 2 and 4 by increasing the stringency of the clustering algorithm (see Methods). Cluster 4 separated in 5 sub-clusters, of which two showed subtle differences in their peak of expression (Fig. 2a, left) i.e. genes in cluster 4A displayed a peak in their expression earlier than those of cluster 4B (Fig. 2b, top). These two clusters represent G_1_/S transition and S phase gene expression respectively as they included several *bona fide* markers of these two phases. G_1_/S regulators, discussed in previous section, indeed belonged to cluster 4A (G_1_/S cluster). This cluster also contained the majority of genes known to be involved in the formation of the pre-replication complex, necessary to initiate DNA replication (*MCM 2-7/10*, *CDC6*, *CDT1* and *ORC1*) [25]. On the other hand, in cluster 4B (‘S phase’ cluster) we identified genes playing a role in DNA replication, particularly in the initiation of DNA replication including cell division control protein 45 homolog (*CDC45*) [25], DNA polymerase alpha catalytic subunit (*POLA1*) and PCNA associated factor (*KIAA0101*). DNA metabolism factors including *RRM1*/*2* were present in cluster 4B, responsible for providing precursors necessary for DNA synthesis.

Likewise, increasing the stringency of the clustering split cluster 6 into two sub-clusters, cluster 6A and cluster 6B (Fig. 2a, right), associated with G_2_ and M phase respectively (Fig. 2b, bottom). Cell cycle regulators *CDK1*, *CCNA2*, *CDC25B* and *CDC25C* were found in cluster 6A (G_2_ cluster) and are known to intervene at the G_2_/M boundary [6]. Kinetochore proteins (*CENPE*, *CENPF*, *CENPI*) and motor proteins (*KIF11*, *KIF14*, *KIF18A*, *KIF18B*, *KIF20B*, *KIF22*, *KIF23*, *KIF2C*, *KIF5B*) were included in this cluster. Cluster 6B was populated with mitotic cyclins such as: *CCNB1* and *CCNB2*, *BUB1*, involved in the metaphase checkpoint [26] and other gene products playing a role in mitosis. Complete description of the phases assigned to genes in clusters 4 and 6 can be found in Additional file 2: Table S1. It should be emphasized however that whilst sub-division of clusters 4 and 6 may identify regions in the network that are more enriched for genes associated with particular phase of the cell cycle, the network is a continuum and these sub-divisions are relatively arbitrary.

### G_1_-related and early growth response clusters

Cluster 2 showed a partial cell cycle-associated expression with peaks of expression coinciding to those of cluster 6 as observed in the following experiments: in experiment three in U2OS cells, in all the three experiments in HeLa cells and in the second experiment in HaCat cells (Fig. 1b, grey profile). Notably, this cluster profile also showed up-regulation at around 6 h in primary fibroblasts entering cell cycle from quiescence (starvation experiment). Genes in cluster 2 were involved in pathways indicative of an active metabolism such as: *cellular metabolic process* (*P* = 2.3 10^−4^), *ribosome biogenesis* (*P* = 3.8 10^−2^) and *macromolecule modification* (*P* = 1.4 10^−2^) (Fig. 1c, grey barplots). Also were found in this cluster: *E2F5*, a member of the E2F transcriptional factors family, which plays a role as repressor during G_1_ phase [27], the retinoblastoma protein (*RB1*), a main tumor suppressor which inhibits cell cycle progression during this phase by inactivating *E2F1* [28] and *CDC73*, another tumor suppressor which has been reported to interact with cyclin D1 [29]. Cluster 2 also included several mitogen-activated MAP kinases (*MAP2K1*, *MAP3K4*, *MAP3K7CL*, *MAP4K3*, *MAPK6*) essential to deliver mitogenic stimuli signals to cell cycle regulators. Interestingly, cluster 1 and 9 (Fig. 1a) also contained G_1_-related genes with cluster 1 including cyclins D1 and D3 (*CCND1* and *CCND3*), master regulators of G_1_ progression [28] while cluster 9 included *CDK4*, a cyclin dependent kinase which operates during G_1_ phase. [28] These clusters however failed to show expression patterns associated with cell cycle events (Additional file 1: Figure S8, green and red profiles). Cluster 3 showed a conserved sharp peak in expression in the first hours after the release of cells from blockade, with no further induction at other times (Fig. 1b, brown profile). The 128 genes in this cluster were highly enriched with pathways involving transmission of both proliferative and anti-proliferative signals (Fig. 1c, brown barplot). Accordingly, the cluster included several genes activated by mitogenic stimuli and encoding for a variety of cytoplasmatic enzymes, secreted proteins and transcription factors assigned to transduce the signal from the cell membrane to the nucleus [30]. These included early growth response genes 2/3 (*EGR2* and *EGR3*), fos and jun (*FOSB* and *JUNB*) which activate transcription upon dimerization [30] and Immediate early response gene 2/3 (*IER2* and *IER3*).

A full list of genes included in the six clusters identified and lists of their enriched GO biological process terms can be found in Additional file 2: Table S1 and Additional file 3: Table S2, respectively.

### Validation of clusters analysis with another unsupervised clustering technique

We analyzed the data using weighted correlation network analysis (WGCNA), an unsupervised technique that generates modules (clusters) of correlated genes after construction of an adjacency matrix. We identified (color-coded) modules after hierarchical clustering using the WGNCA package (see Methods) [17] (Additional file 1: Figure S9A). Reassuringly, comparisons of the genes included in the most enriched modules derived from the WGNCA analysis and genes in the clusters identified with BioLayout *Express* ^3D^ showed high overlap and GO enrichments for each module (Additional file 1: Figure S9B) showed consistency of GO biological process terms, particularly for clusters 2, 3, 4, 6 (Additional file 1: Figure S9C). Moreover, we compared the overlap between the two sets of clusters/modules enriched with cell cycle genes finding 237 genes in common. Though WGCNA analysis identified many other genes included in the two modules (449) (Additional file 1: Figure S9D), the enrichment for the GO_BP term *cell cycle* in the two clusters found in our analysis was far more significant (Additional file 1: Figure S9E).

### A network analysis of the combined data more efficiently identifies commonalities in cell cycle-related genes

**Fig. 3:**
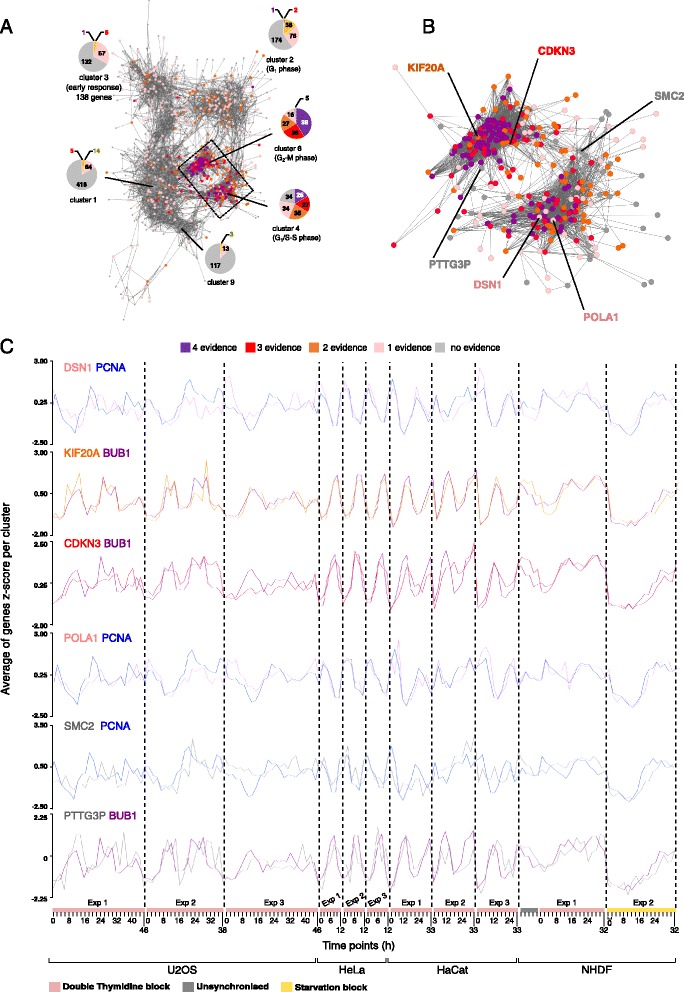
Overlay of cell cycle gene lists from other studies on the graph. (**a**) Nodes from the graph previously clustered were color-coded according to the degree of supportive evidence from published cell cycle gene lists. (**b**) Magnification of the clusters associated with G_1_/S-S phase and G_2_/M phase to show some examples of cell cycle genes found in our analysis but not detected in each of the previous cell cycle studies. (**c**) Expression of six transcripts showing periodic-like expression with superimposed known cell cycle factors. Color scheme in legend refers to all the three sections

We identified 298 cell cycle genes up-regulated during G_1_/S-S and G_2_-M phase across independent studies in different human cell lines whereas direct comparison of the results of individual cell cycle studies showed only 96 common genes. To look deeper at the cause of the poor overlap we overlaid the gene sets from the four studies [6, 7, 9, 10] on the network graph. Notably, the highest overlap was in clusters 4 and 6, representing G_1_/S-S and G_2_-M phases (Fig. 3a). However many genes in clusters 4 and 6 were not reported by all studies with 63 genes identified by three studies, 62 genes by two, 50 genes by one study and 39 genes not reported by any (Fig. 3b). Nevertheless, their expression profiles did show cell cycle-dependent regulation across all the cell lines and many of them are documented to be involved in cell cycle. We illustrate this by describing few examples below. Their relative expression profiles with superimposed known-cell cycle factors can be seen in Fig. 3c. The Kinetochore-associated protein DSN1 homolog (*DSN1*), necessary for proper chromosome alignment and segregation during mitosis as part of the MIS12 complex [31] was only reported in Grant et al. study. *KIF20A*, a mitotic kinesin required for cytokinesis [32], was only found in HaCat and U2OS cells. *CDKN3*, a tumor suppressor phosphatase intervening during G_1_/S transition and mitosis, was not identified by Bar-Joseph et al. study and DNA polymerase alpha catalytic subunit (*POLA1*), essential for DNA replication initiation was only reported by Whitfield et al. study. Genes not supported by any study showing cell cycle-associated expression included structural maintenance of chromosomes protein 2 (*SMC2*), a central component of the condensing complex assigned to condense chromatin into mitotic-like chromosomes [33] and putative pituitary tumor-transforming gene 3 protein (*PTTG3P*), potentially involved in chromosome segregation. A table with complete gene listing of the clusters and the overlap from previous studies can be found in Additional file 4: Table S3. In summary, the majority of the genes in cluster 4 and 6 were not identified in all studies despite following a cell-cycle dependent expression pattern. Thus, correlation-based analysis of the collated data enables bypassing incongruences as a result of the independent analyses and finds coherent patterns in the data.

### Data comparison with yeast data and further human-derived datasets

As a large body of work on cell cycle transcriptomics have been performed on budding and fission yeast, we sought to compare our results with these studies. To do so, we exploited a web resource called Cyclebase (Cyclebase.org) [13] in which several yeast studies were re-analysed and genes were ranked according to the magnitude of their periodicity scores calculated by a statistical method that proved to give the best performance when compared to others [12]. Ranked list of genes were downloaded from the website for the budding and the fission yeasts. The fission yeast list included 449 ranked periodic genes while the budding yeast list comprised a ranked list of the whole yeast transcriptome of which only the top 500 genes were used for comparison. Yeast orthologues to human genes were retrieved using YeastMine (http://yeastmine.yeastgenome.org) for budding yeast, and for fission yeast using PomBase (http://www.pombase.org/). The list of 298 cell cycle-associated genes identified here included 63 orthologues in budding yeast and 35 from fission yeast. When compared with the results of individual human cell cycle studies, the number of budding yeast orthologues is comparable, although in fission yeast almost a double amount of orthologues are identified by the Grant et al. study (Additional file 1: Figure S10A). Nonetheless the 96 genes overlapping in the four studies included a significantly lower number of orthologues genes compared to this list in both yeasts (Additional file 1: Figure S10A). All in all our list includes a relatively high number of orthologues which are mostly comparable with much larger gene lists and a marked higher number than those found in the 96 gene set. To further verify the quality of the 298 gene set we compared its GO enrichments for *cell cycle* biological process term across the four cell cycle gene lists derived from the correspondent individual studies and the set of 96 genes derived from their direct comparison (Additional file 1: Figure S10B). Gene lists from the Whitfield study were obtained both from Cyclebase and from the original study. As it can be seen, our list of genes received the highest enrichment for the GO_BP term *cell cycle*.

## Discussion

Cataloguing the genes involved in the cell cycle has proven to be a challenging task. In human, individual studies have identified highly variable lists of cell cycle-associated genes, with only 96 cell cycle genes being common to all studies performed on different cell types i.e., HeLa, primary fibroblasts, HaCat and U2OS cells [10]. We therefore set out to perform a meta-analysis of a collated datasets to identify modules of genes co-expressing among the four cell types. Specifically, we identified two clusters containing 298 genes, associated with G_1_/S-S, and G_2_-M stages in all the cell types examined which were highly enriched for GO terms associated with early and late cell cycle progression. As circadian rhythm-associated genes are known to oscillate in expression [34], we looked for members of this pathway by mapping the 298 genes to Reactome pathways database [35]. However, no genes involved in the circadian clock were identified, nor known members of this biological process were co-clustered together in the correlation network. Of the genes found in clusters 4 and 6 many have been reported by at least one of the previous four studies, however 39 genes have not been identified previously. After a literature search we found that 18 of these showed supporting evidence to be involved in cell cycle, other 13 were characterized as having non-related cell cycle functions and 8 of them were poorly characterized with no supportive literature (Additional file 5: Table S4). The fact that almost half of these genes have been shown to encode proteins associated with the cell cycle further reinforces the quality of the genes found in clusters 4 and 6. Among them were the Structural maintenance of chromosomes protein 2 (SMC2), a subunit of the condensin complex which is essential for chromosome condensation during mitosis [33], and Putative pituitary tumor-transforming gene 3 protein (*PTTG3P*), a pseudogene member of the hPTTG gene family which were found to be overexpressed in a number of human tumors [36]. The 8 uncharacterised genes were of particular interest as they potentially represent novel cell cycle genes. Two of these, C9orf40 and DNAJC9, were confirmed to be co-expressed with known cell cycle genes as shown in diagrams generated with GeneMania [37] (Additional file 1: Figure S11A-B). Further, DNAJC9 was shown to physically interact with Replication protein A (RPA2), which is involved in DNA replication and repair [38] (Additional file 1: Figure S11B). These genes were also shown to be dysregulated in cancer: C9orf40 was reported to be dysregulated in ovarian carcinoma [39] whereas DNAJC9 was shown to be up-regulated in metastatic cervical cancer in cancer stem cells [40, 41].

Unlike genes involved in the core cell cycle machinery, G_1_ phase-associated genes did not all cluster together in one unique cluster suggesting that this cell cycle phase is less conserved across cell types. G_1_ phase involves cell growth, and therefore it may be more dependent on a given cell type, as cell size and metabolism is highly variable across human cell populations [42]. Of the three clusters containing G_1_ phase genes (cluster 1, 2 and 9), cluster 2 included a significantly higher number of periodic genes supported by two previous studies (38 genes) compared to the other two clusters (cluster 1 = 14 genes, cluster 9 = 3 genes) (Fig. 3a) and represented a pattern of expression consistent with G_1_ phase. This was more pronounced in cells entering proliferation from quiescence as opposed to entering it from a previous cell division, possibly because G_1_ phase in actively cycling cells is shorter than in cells entering proliferation from quiescence [43]. For instance, cyclin D1, responsible for G_1_ progression, is degraded when cells are not actively cycling and has to be newly synthetized upon cell cycle entry. In contrast cycling cells have enough gene product to go through forthcoming cell cycles so reducing the overall time of the cell cycle [28]. However, G1-associated gene expression remains largely elusive. For example, an additional study specifically aimed to identify genes differentially expressed during G_1_ phase in cycling HeLa cells [44] identified 200 transcripts which however did not match any of those found in the four studies nor did they cluster together in our analyses. Further analyses focused on characterizing the G_1_ phase transcriptional regulation will be then of value, especially in view of its crucial role in aberrant proliferation.

Cluster 3 included early growth response genes greatly induced soon after cells were released from the synchronisation block and dropping to basal level for the rest of the experiment. This suggests that this set of genes might be essential only in triggering proliferation from a cell cycle arrest or quiescence (induced by drugs-based﻿ or serum starvation synchronisation methods, respectively) but are not needed to induce a second cell cycle in actively cycling cells. The presence of this cluster is yet another clue that the transcriptional regulation of cells entering cell cycle from quiescence compared to cycling cells is significantly different.

Finally, we note that 298 is a highly conservative estimate of conserved cell cycle genes. In collating datasets derived from different microarray platforms, thousands of genes were discarded leading to only 11,693 unique Entrez ID entries (approximately half of total number of the human genes) being shared across platforms.

## Conclusions

These findings suggest a far more conserved transcriptional network associated with the human cell cycle than might be suggested by just comparing previous gene lists, which is in line with this system to be highly conserved across evolution. Moreover, additional biologically relevant clusters were found using such exploratory analysis, free from a priori hypothesis. The limited number of shared cell cycle genes reported previously is therefore likely to be primarily due to analysis protocols, similar to the conclusions drawn on meta-analyses on budding and fission yeasts studies [11, 12]. We also speculate that the number of conserved cell cycle genes might be even higher given the intrinsic limitations of this analysis approach.

## Additional files

Additional file 1: Figures S1-S11. Supplementary figures. (DOCX 5416 kb)
Additional file 2: Table S1. Lists of genes included in the clusters. (XLSX 52 kb)
Additional file 3: Table S2. GO enrichment analyses of the clusters. (XLSX 1180 kb)
Additional file 4: Table S3. Overlay of previous cell cycle gene lists on the clusters. (XLSX 75 kb)
Additional file 5: Table S4. Table S4. Annotation of the 39 unreported genes. (XLSX 10 kb)
