# Supplementary material for: Meta-analysis reveals conserved cell cycle transcriptional network across multiple human cell types
Source: BMC Genomics. 2017 Jan 5;18:30. doi: 10.1186/s12864-016-3435-2 (PMC5217208; doi:10.1186/s12864-016-3435-2)
Supplement: Additional file 1: Figures S1-S11. — Supplementary figures. (DOCX 5416 kb) [file 12864_2016_3435_MOESM1_ESM.docx]

**Supplemental Figures**

**
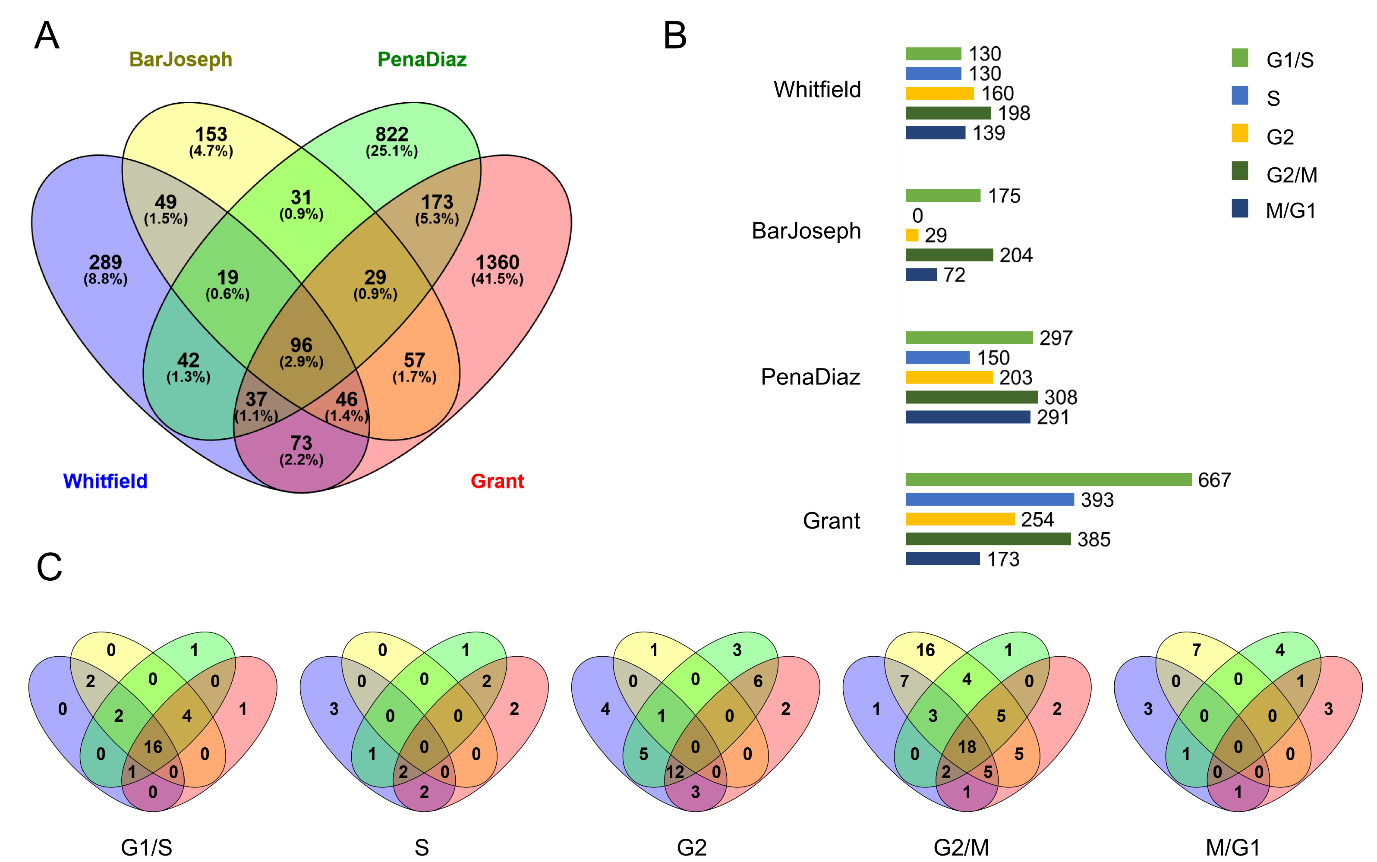
**

**Figure S1. Overlap of previous cell cycle gene lists and overlap of their phase assignations.** (A) Venn Diagram of the cell cycle genes (unique Entrez IDs) identified in four previous cell cycle studies. (B) Size of phases assigned to cell cycle genes per cell cycle study. (C) The 96 genes shared across the four studies are further overlapped based on their phase assignation.

**
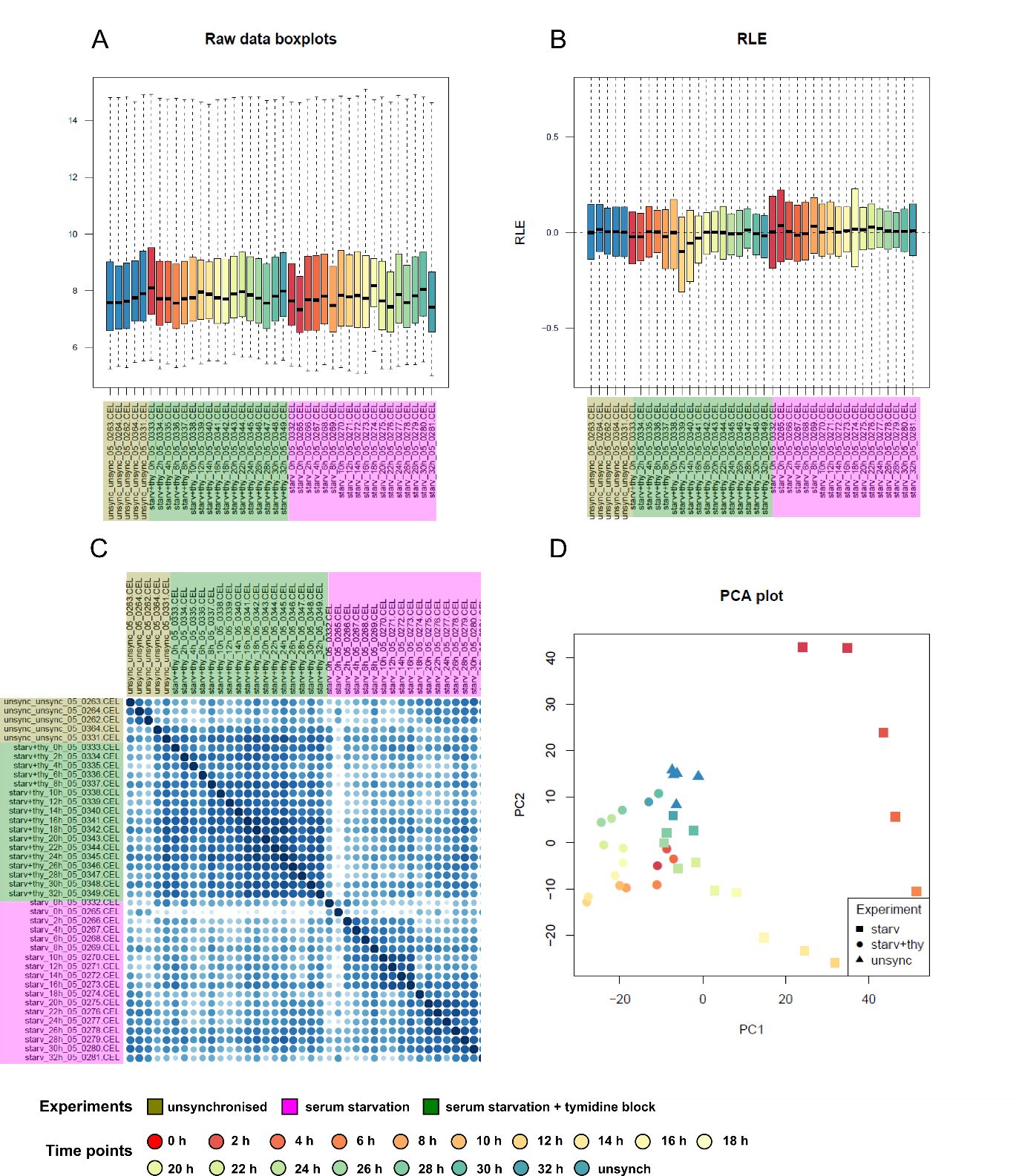
**

**Figure S2. Quality control tests for Bar Joseph et al. data.** Arrays were tested for three metrics: (**A**) Boxplots of raw signal intensities, (**B)** Relative log expression (RLE) and (**C**) Sample to sample correlation matrix (**D**) PCA plot of Bar-Joseph et al. data showed separation by experiment in accordance with cells synchronised at different cell cycle phases. Clustering by time point was present too for both experiments. Later time points are in proximity of control samples (not-synchronised). Samples are colored by time-point and experiments are highlighted with different colors (different symbols in PCA plot) (see legend).

**
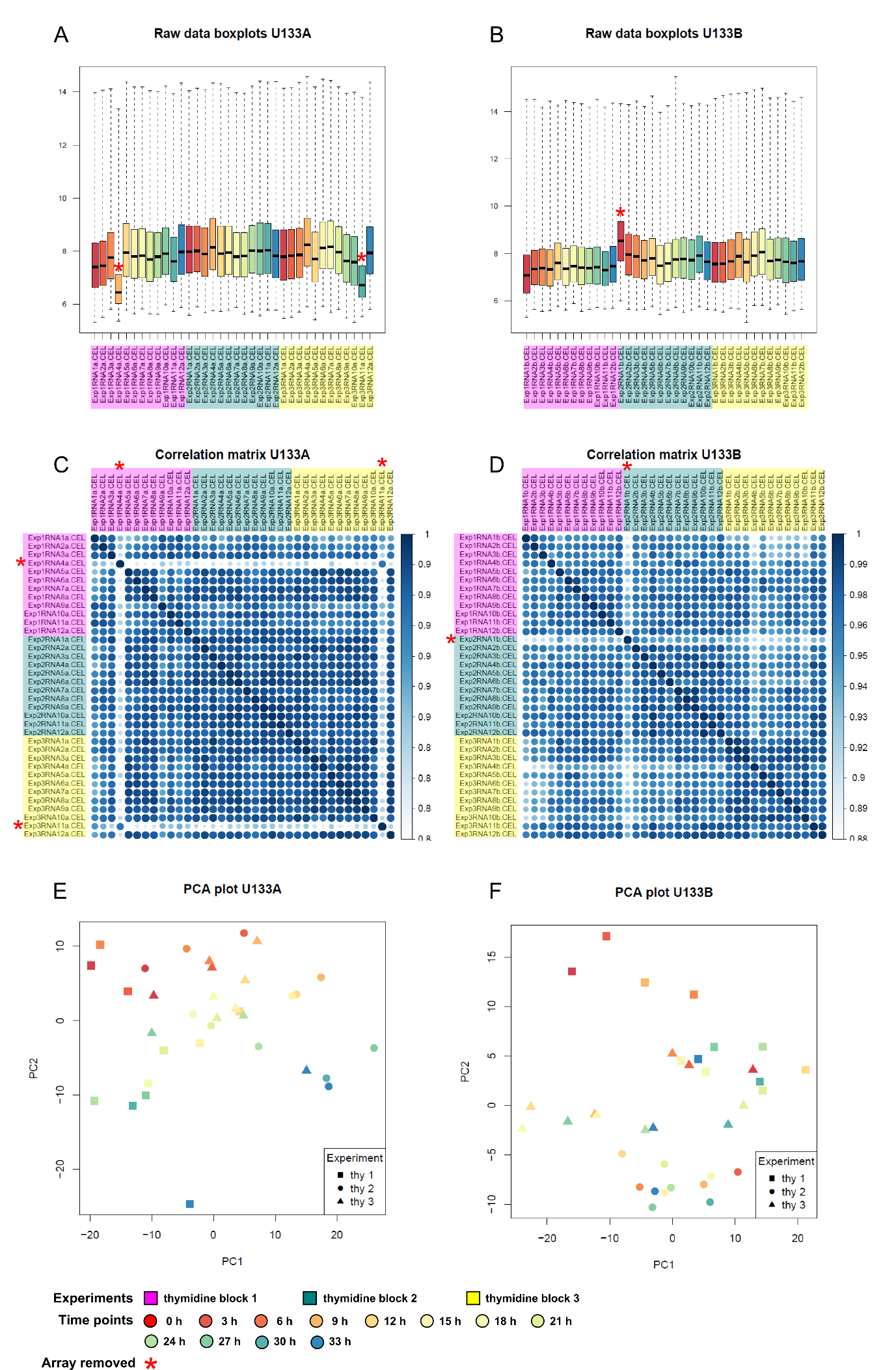
**

**Figure S3. Quality control tests for Pena-Diaz et al. data** Arrays were tested for two metrics. RLE plot was not available for this platform: (**A-B**) boxplots of raw signal intensities for chip U133A and U133B. (**C-D)** Sample to sample correlation matrix for chip U133A and U133B. (**E-F**) PCAs of the complementary chip sets U133A and U133B. Partial clustering by time point is visible for U133A PCA plot while U133B PCAs showed a separation driven by experiment. Samples not passing the quality tests are marked with a red asterisk. Samples are colored by time-point and experiments are highlighted with different colors (different symbols in PCA plot) (see legend).

*
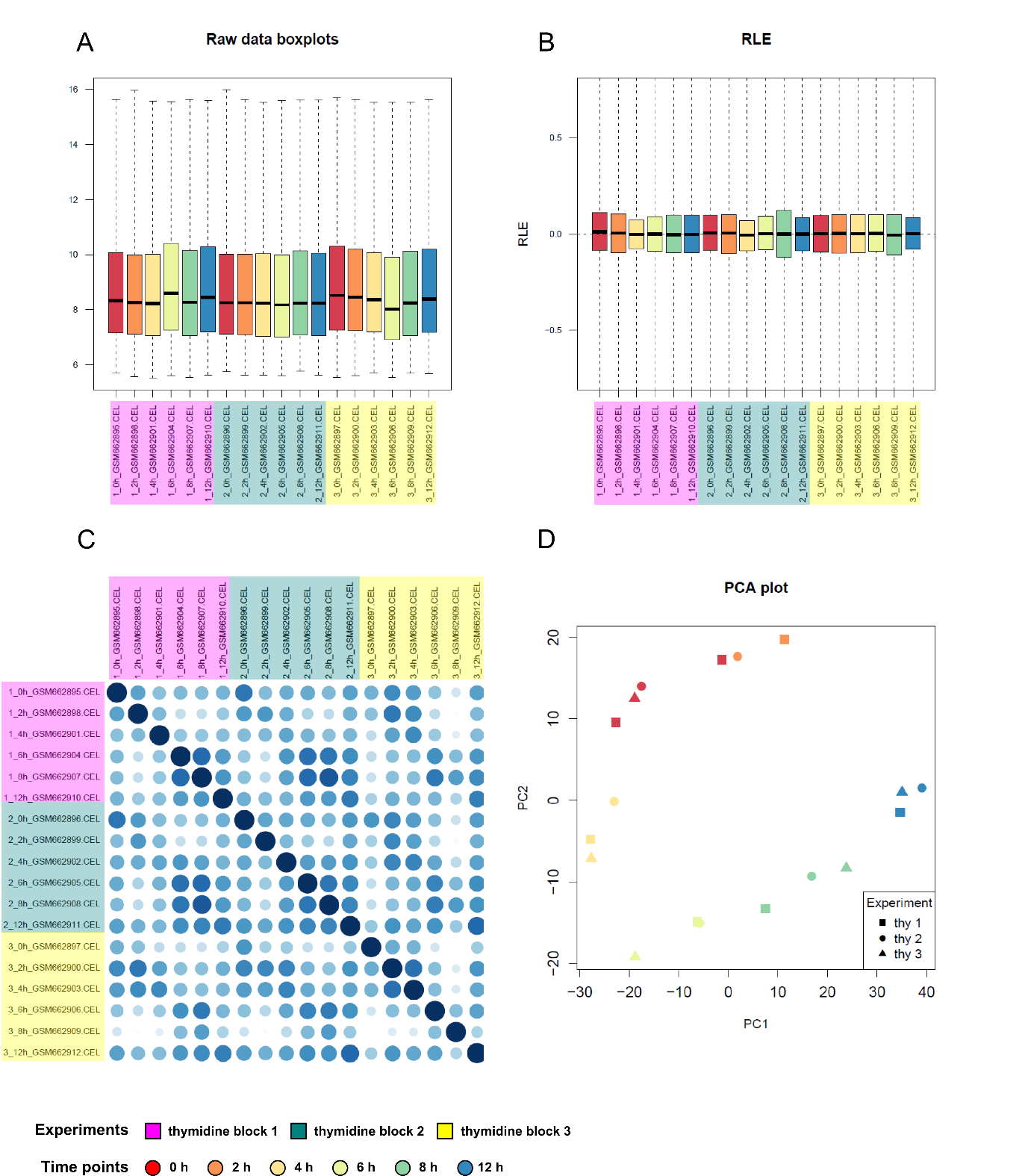
*

**Figure S4. Quality control tests for Sadasivam et al. data.** Arrays were tested for three metrics: (**A**) Boxplots of raw signal intensities. (**B**) Relative log expression (RLE). (**C**) Sample to sample correlation matrix. (**D**) PCA plot showed neat clustering by time point across the three biological replicas. Samples not passing the quality tests are marked with a red asterisk. Samples are colored by time-point and experiments are highlighted with different colors (different symbols in PCA plot) (see legend).

***
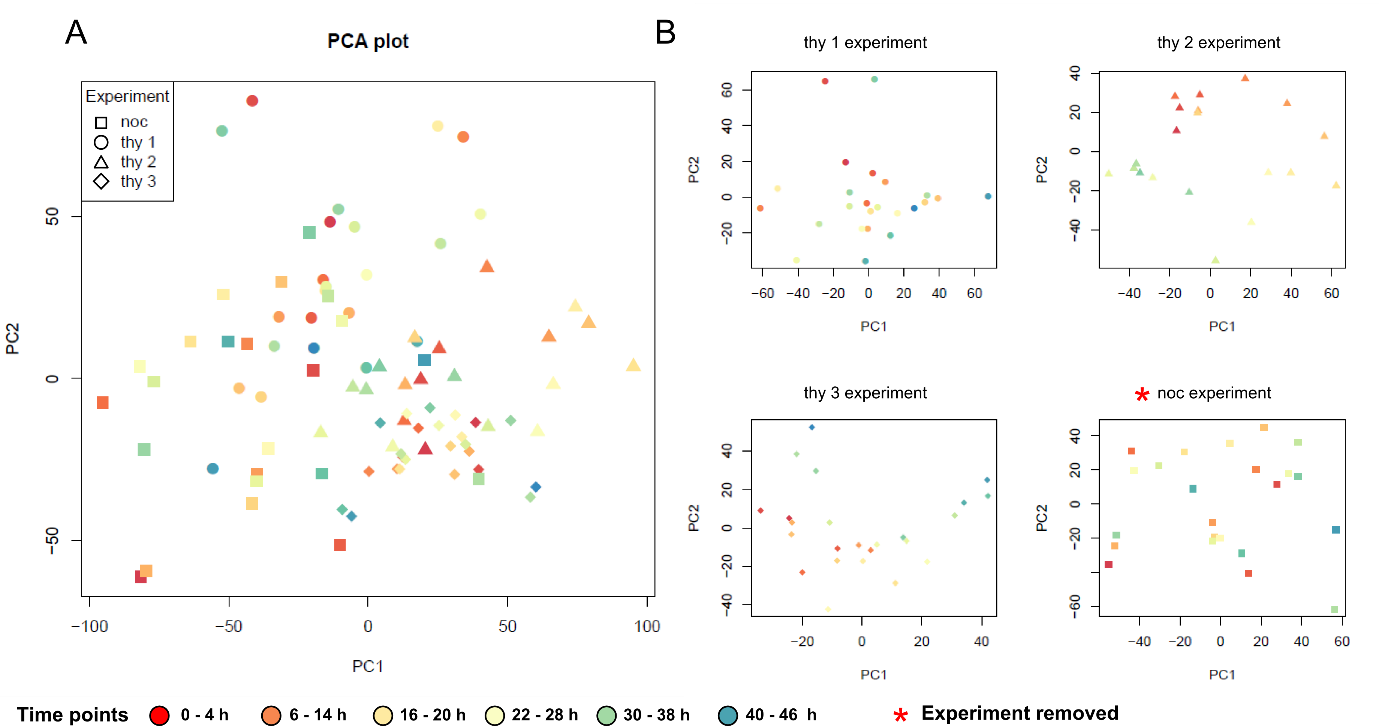
***

**Figure S5. PCA plots of Grant et al. data.** (**A**) PCA plot of the whole dataset showed no clustering by any factor. (**B**) The four experiments are plotted separately and a partial clustering by time point can be seen. The nocodazole experiment (noc experiment) however still did not show any clustering and for this reason was removed.


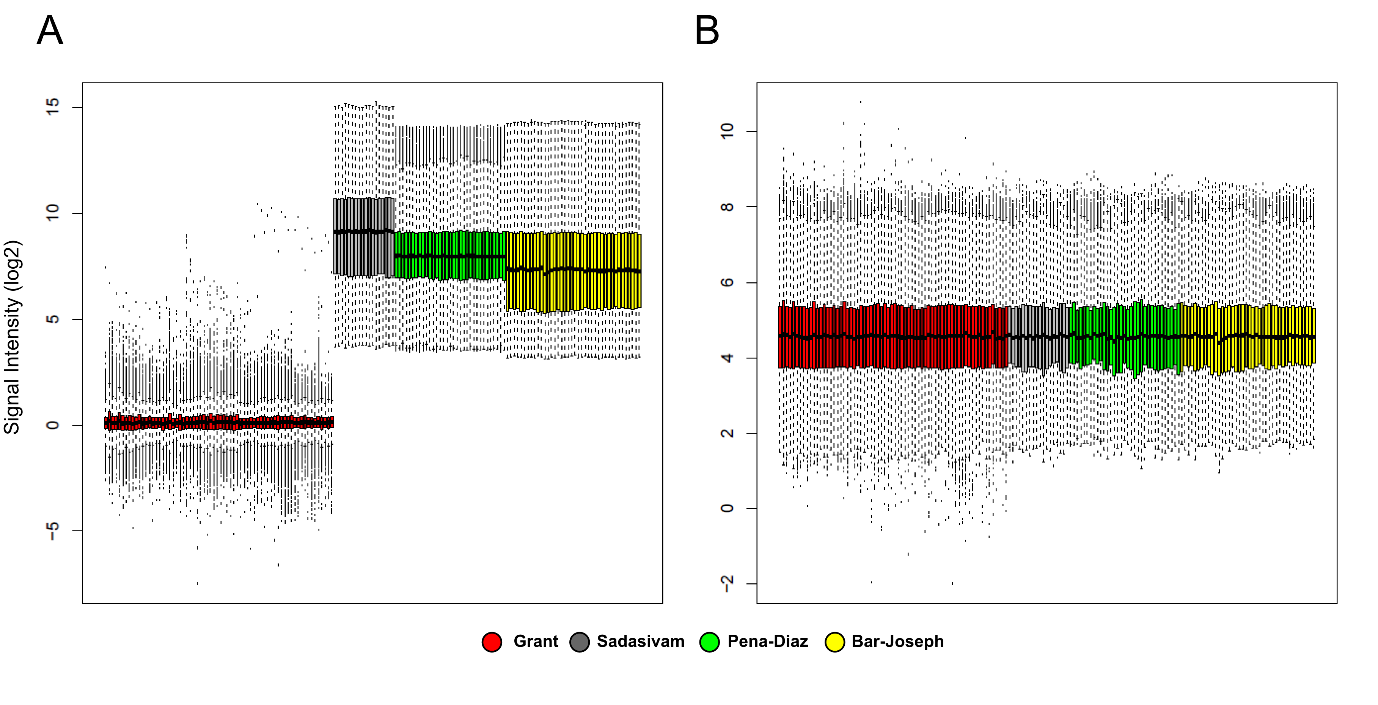


**Figure S6. Batch correction**. (**A**) Boxplots of the four studies. Large differences in signal mean intensity and distribution are evident particularly because Grant data was provided in log ratios of the intensities while for the other three studies single channel microarrays were employed. (**B**) After the batch correction using ComBat mean intensity and distribution were even out.


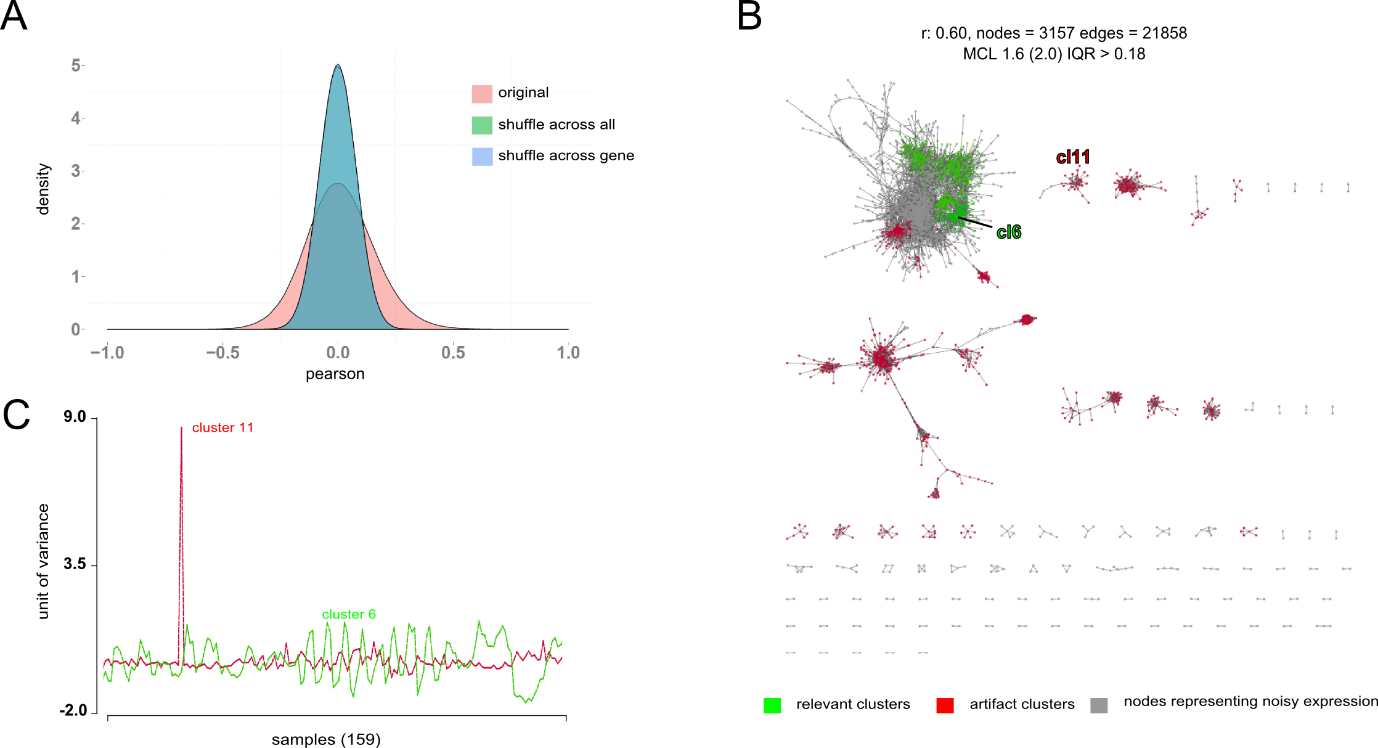


**Figure S7 Generation of the network graph and selection of biologically relevant clusters.** (**A**) Histogram representing the correlation of 2000 random genes from the collated dataset compared with their correlation after measurements were shuffled across genes and across all. As it can be seen the probability of having two or more genes randomly correlated is less than original data and the threshold set for analysis (r ≥ 0.60) is well above it. (**B**) The graph included 3157 nodes of which 785 were falling in four clusters, colored in green, considered of relevant biology to cell cycle. In contrast clusters showing artifact expression were colored in red and accounted for 722 nodes. The rest of the nodes were colored in grey and represented noisy/ non-relevant-to-cell cycle expression (**C**) Example of clusters showing artifact expression (cluster 11) or real expression (cluster 6). Cluster 11 showed a sharp peak on a single sample not repeated in the replicas suggesting this to be a technical artifact. Cluster 6 showed coherent expression across data.


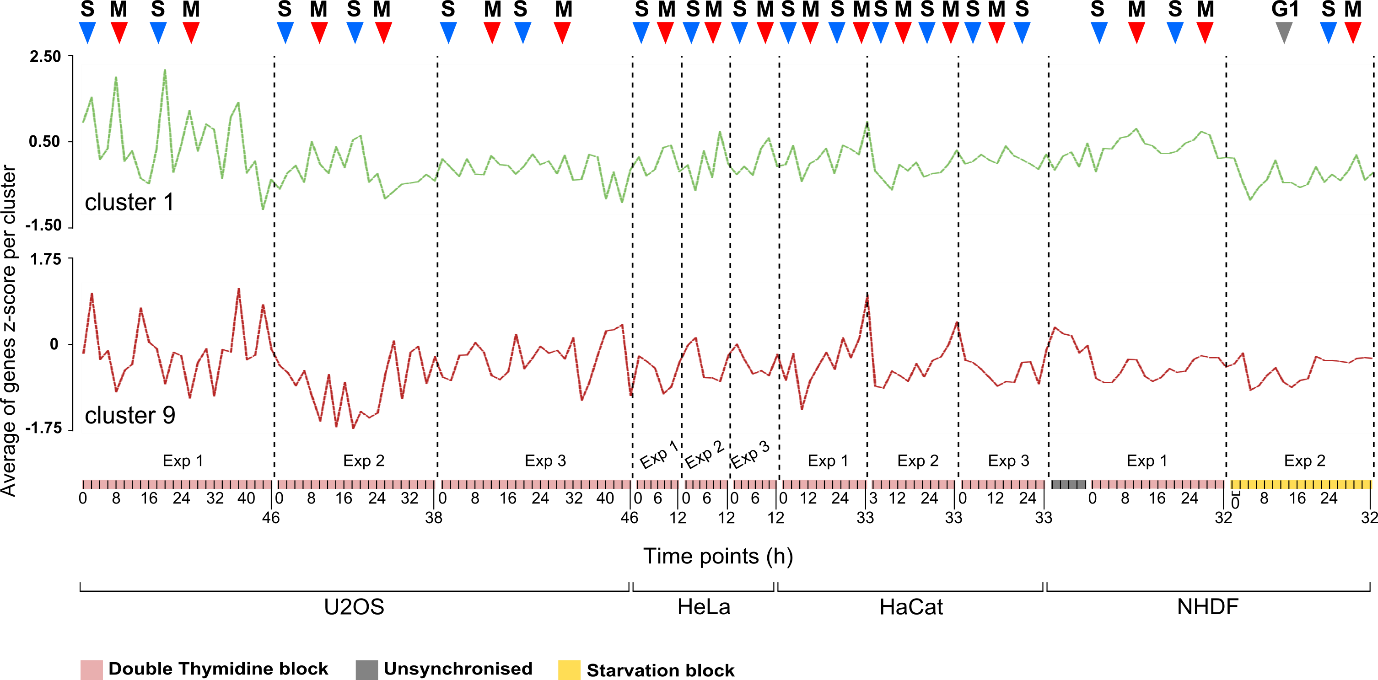


**Figure S8 Clusters profiles not showing reproducible pattern of expressions but including important cell cycle genes.** Cluster 1 and 9 included multiple G1-associated genes. However, their cluster expression profiles did not show reproducibility across samples.


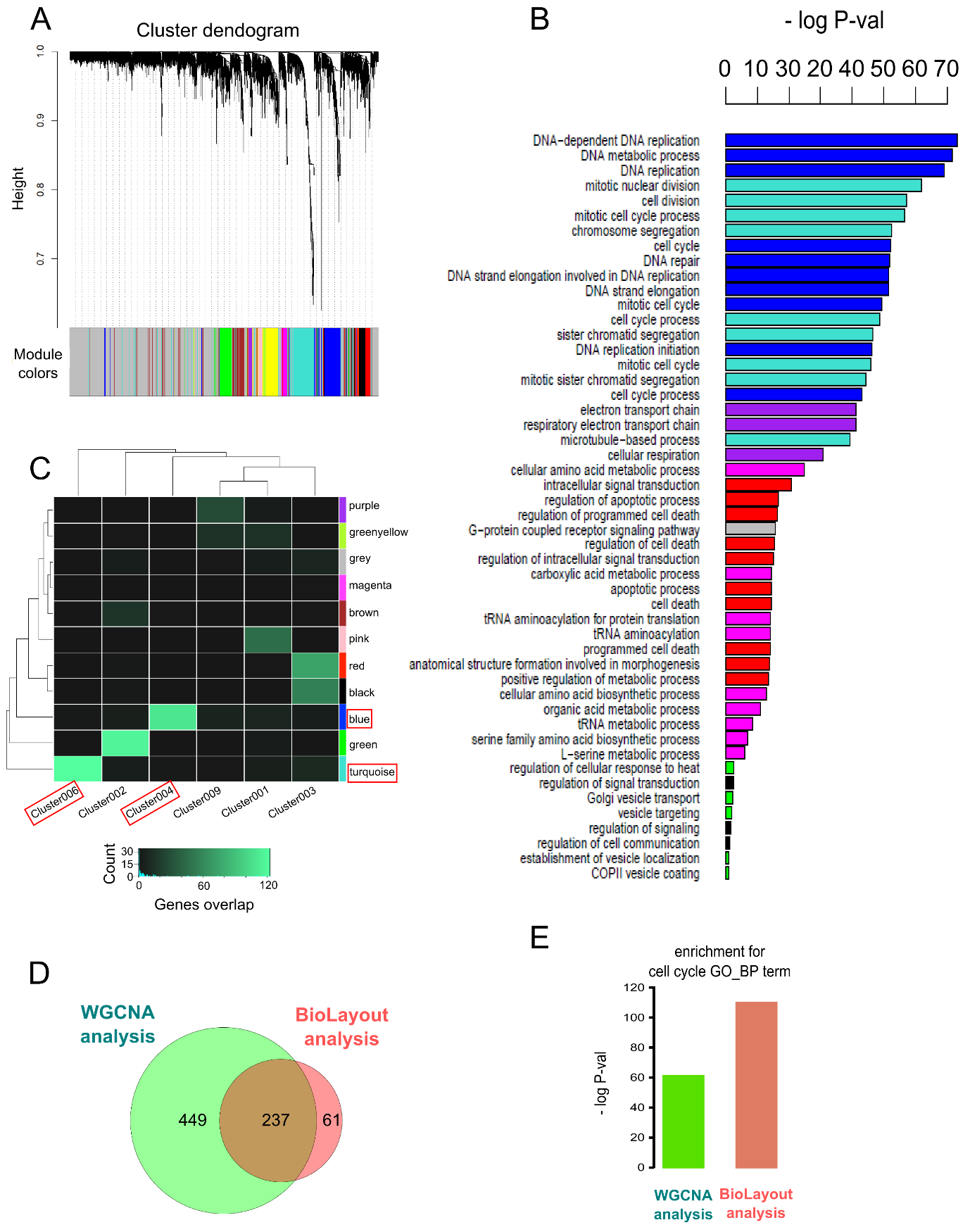


**Figure S9 WGCNA analysis.** (**A**) Modules generated with hierarchical clustering after running the function blockwiseModules. Colors were associated to each module. (**B**) GO enrichment performed on the modules. Only the most enriched modules are shown of which the top two were the blue and the turquoise modules, respectively enriched with S phase and M phase-related biology. (**C**) Overlap of the genes found in the modules with WGCNA and with BioLayout Express^3D^_._ High overlap of the clusters/modules enriched with cell cycle terms was observed. (**D**) Venn Diagram of the genes contained in the two modules and in the two clusters enriched for cell cycle terms derived from correspondent analyses. (**E**) Enrichment for the GO_BP term cell cycle showed a far higher enrichment for the two clusters identified in our analysis.


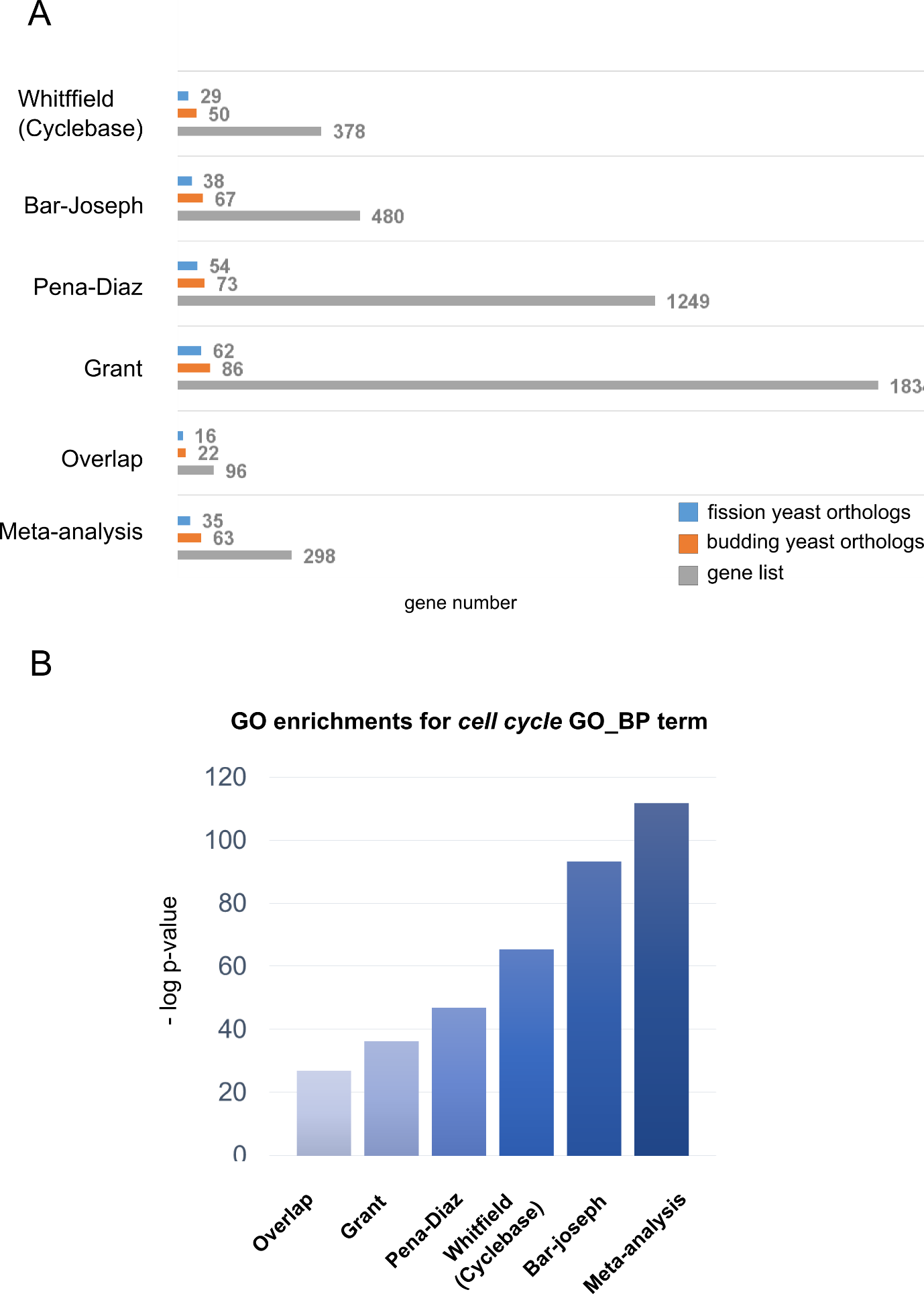


**Figure S10 Yeasts orthologues of periodic genes and GO enrichment comparison with previous cell cycle studies.** (**A**) Budding and fission yeasts periodic genes retrieved from Cyclebase were compared with periodic genes from human studies. Barplots shows that our list (meta-analysis) includes a comparable number of budding yeast orthologous genes compared with individual human studies and three times more compared with the 96 common genes from direct comparison of the single studies (Overlap). The meta-analysis retained also a higher amount of fission yeast orthologues when compared with the 96 common genes. (**B**) Our list (meta-analysis) received a higher enrichment for the GO Biological Process cell cycle term after performing a GO enrichment on the four individual lists plus the 96 genes set shared across the four studies (Overlap).

**
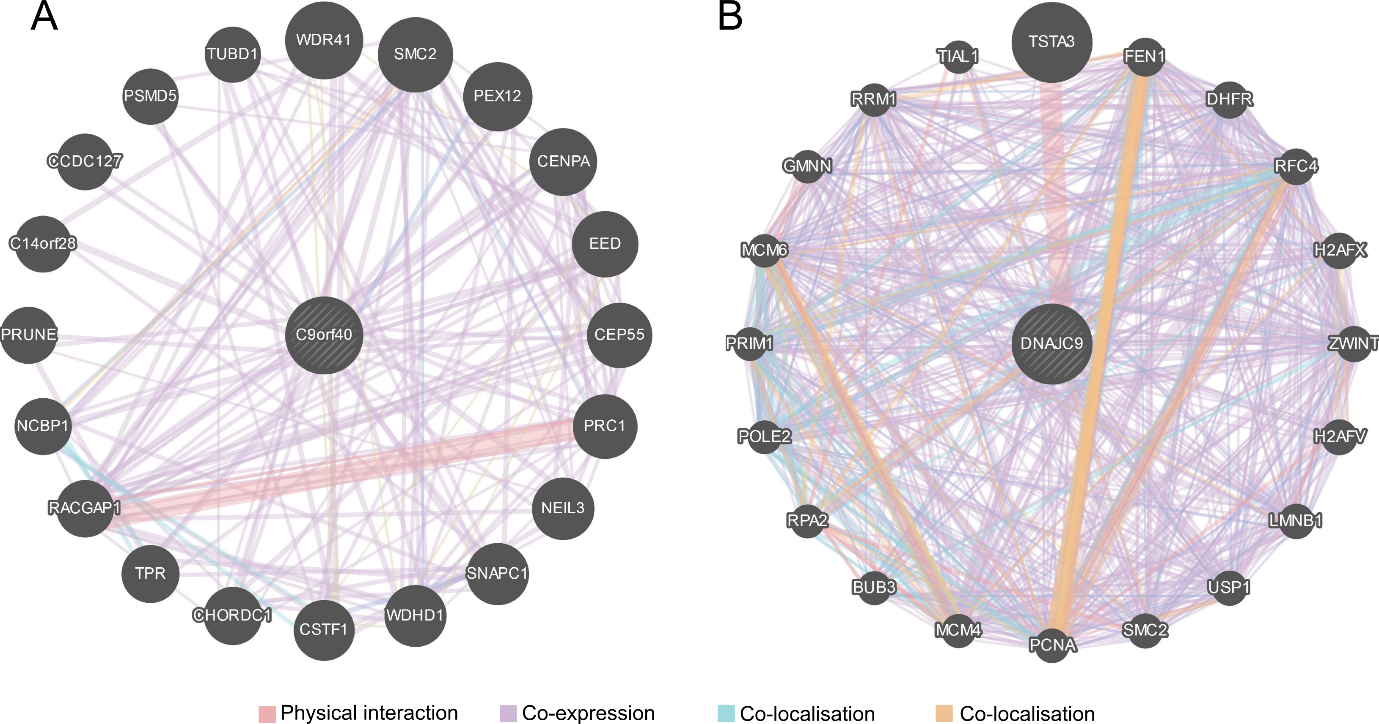
**

**Figure S11 Gene/Protein relations of the uncharacterised genes C9orf40 and DNAJC9.** Gene/Protein to gene/protein relations networks generated using GeneMania of the uncharacterised gene (**A**) C9orf40 and (**B**) DNAJC9. Different colors correspond to difference evidence as shown by the legend at the bottom.
